# Participatory monitoring in community-based fisheries management through a gender lens

**DOI:** 10.1007/s13280-022-01783-3

**Published:** 2022-09-20

**Authors:** Jenny House, Danika Kleiber, Dirk J. Steenbergen, Natasha Stacey

**Affiliations:** 1 Research Institute for the Environment and Livelihoods, Charles Darwin University, Casuarina, NT 0810 Australia; 2 Pacific Islands Fisheries Science Center, National Marine Fisheries Service, National Oceanographic and Atmospheric Administration, 1845 Wasp Blvd, Honolulu, HI 96818 USA; 3 Australian National Centre for Ocean Resources and Security, University of Wollongong, North Wollongong, NSW 2500 Australia

**Keywords:** Critical interpretive synthesis, Local knowledge, Small-scale fisheries, Systematic review, Women’s participation

## Abstract

In small-scale fisheries management, the significance of participation is widely recognised but we are still learning how this can be better operationalised to include different groups, such as women or Indigenous peoples. Participatory monitoring is one tool which has been used to increase participation in fisheries management. The aim of this review is to use critical interpretive synthesis to examine the literature on participatory monitoring within community-based fisheries management from a gender perspective. The synthesis identified and discussed several key areas: reasons presented in the literature for engaging with the themes of gender or participatory monitoring, gendered aspects of participatory monitoring, knowledge valuation and prioritisation in management, replicability and transparency of programme or research methods, and marginalisation narratives. Our findings show the complexities of conducting gender-aware participatory monitoring. Participatory monitoring has the potential to be a transformative and empowering process if the power dynamics involved are considered and addressed.

**Supplementary Information:**

The online version contains supplementary material available at 10.1007/s13280-022-01783-3.

## Introduction

Community-based and co-management arrangements are increasingly being used to manage small-scale fisheries (SSF) (Jupiter et al. 2014; Cohen and Steenbergen 2015). These approaches to management are part of a growing trend towards decentralisation, which prioritises the involvement of resource users and local communities (FAO 2015). SSF are usually characterised as diverse, complex, dynamic, labour-intensive, multi-species, multi-gear fisheries (Johnson 2006; Mills et al. 2011; Smith and Basurto 2019). Over 90% of the world’s fishers are involved in SSF (The World Bank 2012), and 97% of these people live in developing countries (Mills et al. 2011). With so many people relying on SSF for food (Thompson et al. 2002; Bell et al. 2009; The World Bank 2012), livelihoods (Allison, 2004; Smith et al. 2005) and cultural identity (Poepoe et al. 2003), sustainable management of SSF is critical. The 2015 FAO Voluntary Guidelines for Securing Sustainable Small-scale Fisheries (SSF Guidelines), advocate for greater consultation and inclusion of local communities, Indigenous peoples, and women in the SSF sector. Participatory monitoring is one tool which has been used in fisheries management to collect data and increase participation of historically excluded groups in the decision-making process.

Gender equality and women’s participation are now seen as an important part of small-scale fisheries management (FAO 2015; Lawless et al. 2021). Nevertheless, the contribution of women to fisheries has often been overlooked and is poorly understood (Kleiber et al. 2014; Harper et al. 2017). Women often participate in different parts of the SSF value chain (Overa 1993; Pedroza-Gutiérrez 2019), fish in different ways from men (Chapman 1987; Kleiber et al. 2014), and have different knowledge about marine resources (Siar 2003; James et al. 2021). Therefore, including women in management activities may improve natural resource management outcomes (Leisher et al. 2016, 2017). To elucidate these distinctions, a gender lens is required when engaging with SSF management (Williams 2008). Looking at SSF through a gender lens (Box 1) means making gender visible in every aspect of fisheries activities and value chains, and examining the social relations which shape gender inequalities in the fisheries sector. Although our intersectional approach acknowledges and explores the overlapping social characteristics, such as race, class, or age, which impact people’s participation (Rice et al. 2019), our primary focus in this study will be on gender.

Box 1: Sex and gender Sex and gender are related but different. Sex refers to biological attributes which are used to categorise people as female, male or intersex. Gender, rather than being biological, refers to the social attributes, roles, activities, and responsibilities which are associated with being men and women in a particular community at a given time. These are determined by social norms, power, and institutions. There are a wide variety of gender identities between and beyond the binary of men and women.

There are several authors who deconstruct the conventional binary view of gender in the context of fisheries (Davis and Nadel-Klein 1992; Bennett 2005; Frangoudes et al. 2019). However, much of our discussion relates to a binary view of gender, as our focus is on approaches presented in the literature. Furthermore, gender research in fisheries is often focused on women’s representation or empowerment (as opposed to men’s), due to women’s involvement in the sector being historically overlooked (Kleiber et al. 2014). Our analysis will reflect this emphasis, but we recognise that a comprehensive analysis of gendered aspects of fisheries also requires an understanding of fishers’ masculinities (Gustavsson and Riley 2019; Siegelman et al. 2019) and all historically marginalised genders.

Participatory monitoring is one tool which has been used to increase women’s participation and transform power dynamics in fisheries management (Carvalho et al. 2009; Paul et al. 2016). Participatory monitoring is distinct from other forms of local knowledge. Fishers’ knowledge may consist of observations about fisheries or their experiences (Box 2), but participatory monitoring involves collecting detailed information with the goal of capturing temporal change and using that data to make management decisions. Participatory monitoring can also include community members who are not (or do not consider themselves) fishers. The extent to which these forms of knowledge are integrated into management can range from data being sampled by others or provided by fishers, up to participatory governance where local knowledge is used and valued (Stephenson et al. 2016).

Box 2: Knowledges and participatory monitoring Various terms have been used in the literature to refer to the “experiential knowledge … that fish harvesters accumulate while operating in their respective fisheries” (Hind 2014), such as local knowledge, fishers’ ecological knowledge, fishers’ knowledge, participatory research, observations from the fishery, fishery-dependent data (Stephenson et al. 2016). Fishers knowledge may also include Indigenous knowledge (Deepananda et al. 2015; Ban et al. 2017). Indigenous knowledge is the knowledge which is held and continually developed by Indigenous people, so there is overlap between fishers’ knowledge and Indigenous knowledge. Participatory monitoring may use these forms of knowledge, as well as the local knowledge of non-fishers.

In this paper, we use the term “local knowledge” to refer to fishers’ and non-fishers’ local knowledge, including Indigenous knowledge. The term “Indigenous knowledge” does not include other forms of local knowledge.

The purpose of participatory monitoring is often multifaceted. It can be an effective data collection tool in data-poor systems, as well as point of entry to engage user groups in management. Participatory monitoring is known to enhance engagement (Obura et al. 2002), to enable people to make informed decisions, and can be an empowering process in itself (Freire 1970). Participatory monitoring can enable community members to respond to their own interests and concerns through several parts of the monitoring process; from collecting, analysing, and understanding data, through to decision-making (Obura 2001; Guijt 2007; Evans and Guariguata 2008; Danielsen et al. 2009). Many small-scale fisheries are data-poor so incorporating participatory monitoring and local knowledge (Box 2) into management can inform and improve management (Wiber et al. 2004, 2009). However, efforts to use participatory monitoring approaches and integrate local knowledge into fisheries management are not without risk (Nadasdy 1999). Participatory monitoring and community-based management alone will not shift fisheries management from a system of knowledge assimilation to one of knowledge coexistence (Reid et al. 2020).

Participatory monitoring programmes in fisheries have tended to focus on male fishers collecting catch data (Obura, 2001; May, 2005; Ernst et al. 2010; Prescott et al. 2016). However, as women’s involvement in fishing is increasingly recognised and the potential benefits of participatory monitoring become clearer, monitoring projects which involve women are being established (Aswani and Weiant 2004; Carvalho et al. 2009). Although monitoring programmes may increase women’s participation, Agarwal (2001) shows that being asked to participate in specific tasks (activity-specific participation) does not necessarily result in an empowering process or being able to influence community decisions (Box 3). Organisations which make use of the rhetoric of participation or being community-based, without examining the gender implications of their work, may unintentionally entrench inequality or create new barriers to women’s empowerment (Baker-Médard 2017). Given that participatory monitoring alone does not ensure inclusive fisheries management, it is necessary to reflect on how participatory monitoring has been implemented and presented to fisheries managers and researchers. This reflexivity can be an opportunity to develop participatory monitoring approaches which move beyond activity-specific participation (Agarwal 2001) and become empowering and transformative.

Box 3: Participation in natural resource management Several typologies have been produced to show the different processes of participation and examine how participants are interacting with external actors or each other in a NRM or other context. Some of these emphasise the power dynamics between the community and external actors (Arnstein 1969), whilst others focus on the intracommunity dynamics between groups (Agarwal 2001). This perspective recognises that communities are not homogenous and a person’s ability to participate in decision-making may be influenced by a variety of factors, such as their gender or socio-economic status. Agarwal (2001) describes six levels of participation which range from nominal participation, e.g. membership in the group, to empowering participation, where participants have a voice and are able to influence decision-making. There are various activities involved in empowering participation. These are attending and understanding activities, sharing views, being understood and valued, making decisions and accountability (Kleiber et al. 2019).

In this paper, we will examine the literature on community-based fisheries management (hereafter, CBFM) and small-scale fisheries co-management through a gender lens and explores how participatory fisheries monitoring is represented and used. A critical interpretivist synthesis reveals the extent to which participatory monitoring and gender are considered, and the nature of this engagement. We ask the following questions:

1. How and why do researchers engage with the themes of gender and participatory monitoring within CBFM?
2. How is participatory monitoring in CBFM portrayed and how is it seen to promote and/or compromise participation for women and men within a community?
3. How are the impacts of participatory monitoring on CBFM decision-making processes and activities, and for the participants themselves, characterised in the literature?

We answer these questions through a systematic review of the literature on CBFM in developing countries and conduct a gender analysis of the literature on participatory monitoring within CBFM. Our findings provide guidance on designing and implementing gender-inclusive participatory monitoring programmes and suggest how these themes can be better represented in scholarly outputs and reporting. A more nuanced understanding of these issues will contribute towards developing more equitable and sustainable approaches to fisheries management.

## Method

The aim of this review was to examine how the themes of gender and participatory monitoring are represented in the CBFM literature, rather than to evaluate the CBFM programmes themselves. Critical interpretive synthesis (Dixon-Woods et al. 2006; Bales and Gee 2013) was the primary analysis method. Additional quantitative techniques were used to examine how much of the literature engaged with selected themes related to monitoring and gender.

### Literature search and selection

Documents were included which focused on small-scale fisheries in terms of how community-based management or co-management is done, rather than focussing on the economic or biological elements of the fishery. Papers also had to be research articles that were available in English. However, we acknowledge that overlooking non-English literature can create bias in systematic literature analysis (Konno et al. 2020), may ignore valuable context-dependent evidence (Amano et al. 2021), and can contribute to a Western-biased synthesis of the literature. Documents such as conference proceedings or book chapters were excluded. The search was limited to the year 2000 onwards to increase the relevance of the review’s findings. Research about developed countries was excluded, based on the UN M49, though Non-Self-Governing Territories were included (United Nations and Decolonization 2020; United Nations Statistical Division 2020). Gender was not included as a search term in the initial search because it is recognised that management or research which does not address gender issues explicitly can still have a gendered impact. Understanding the content of gender-blind literature is equally relevant to the research questions as literature which primarily focuses on gender.

**Fig. 1:**
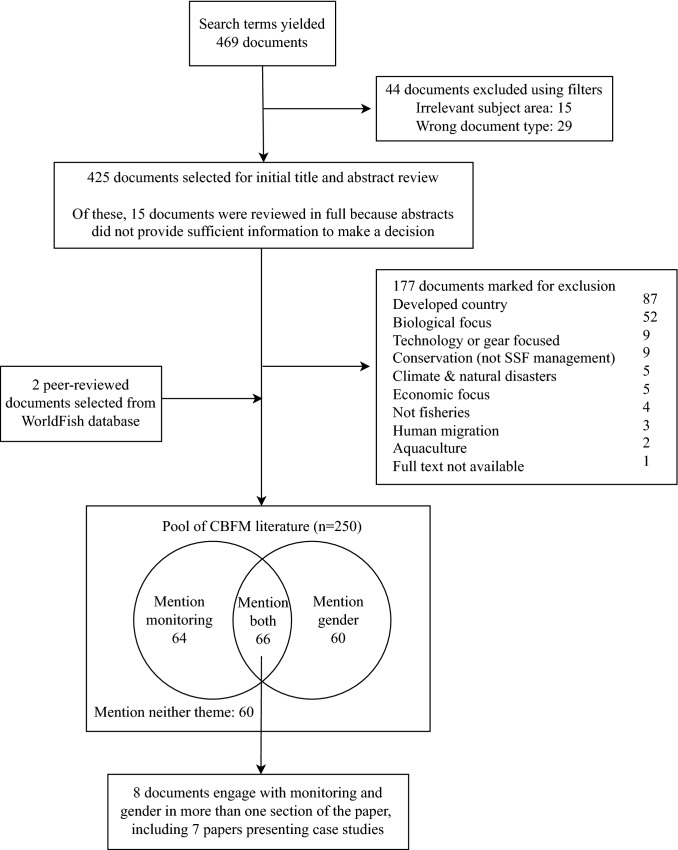
Peer-reviewed literature selection process

As stated in the introduction, there is not a clear definition for small-scale fisheries. In their 2019 systematic review, Smith and Basurto provide suitable search terms for identifying these fisheries. Additional terms were added to ensure that gleaning was included, as this is often a fishing activity dominated by women (Kleiber et al. 2014). In the Web of Science database, the search terms “fisheries”, “co-management”, “community-based”, “small-scale”, “artisanal”, “fisher folk”, “fishing community”, “subsistence”, “gleaning”, “shellfish”, “inshore”, “intertidal” and “gather” were used to find literature; see Table 1 for exact search terms. These terms were used to select community-based management or co-managed small-scale fisheries. The literature search was carried out in December 2020 and repeated in February 2021. This search yielded 469 documents, of which 44 were removed using filters (Fig. 1). A total of 425 documents were selected for title and abstract review, using Rayyan (Ouzzani et al. 2016), to be compared against the inclusion and exclusion criteria. The documents were then screened based on the title and abstract, or the full text if the abstract did not provide sufficient information to decide. A further 177 documents did not meet the inclusion criteria. The result of this process was a pool of literature examining management activities and governance processes within CBFM.

**Table 1:** Literature selection and search terms

Goal of search	Where to search	Search terms
Create pool of literature about the management processes within community-based and co-manage fisheries (hereafter, referred to as CBFM pool)	Web of Science database	(TS=(community-based OR co-management) AND PY=(2000-2020) AND TS=("small-scale fisher*" OR "artisanal" OR "fisher folk" OR "fishing community" OR "subsistence" OR "glean*" OR "shellfish" OR "inshore" OR "intertidal" OR "gather*") AND TS=(fisher*)) AND LANGUAGE: (English)
	WorldFish database	(community-based OR co-management) AND (fishery OR marine resource) Language = English Year = 2000–2020 NB: Limited characters available for search terms so “marine resource” was used to encompass gleaning
	SPC Bulletins: - Women in Fisheries - Fisheries Newsletter - Traditional Management Bulletin	("co-management" OR "community-based") AND fisher* AND ("small-scale fisher*" OR "artisanal" OR "fisher folk" OR "fishing community" OR "subsistence" OR "glean*" OR "shellfish" OR "inshore" OR "intertidal" OR "gather*")
Determine how many CBFM/comanagement papers discuss gender	CBFM literature pool, NVivo	Gender OR Women
Determine how many CBFM/comanagement papers discuss participatory monitoring	CBFM literature pool, NVivo	"participatory monitoring" OR "community-based monitoring" OR "fisher knowledge" OR "ecological knowledge" OR "local knowledge" OR "participatory research" OR "fishers' data" OR "collaborative research" OR "fishery dependent data" OR "cooperative research"

In addition to the peer-reviewed literature, grey literature was selected from several sources. These sources were the WorldFish database (which also provided two peer-reviewed articles) and several of The Pacific Community (SPC) bulletins; *Women in Fisheries, Fisheries Newsletter*, and *Traditional Management Bulletin*. Slightly different search terms were used due to the constraints of each database (Table 1), but the inclusion and exclusion criteria remained the same. Grey literature was not incorporated in the quantitative analysis. However, purposive sampling of these documents was used to complement the qualitative analysis of the peer-reviewed material.

We recognise the limitations of this study, based on the nature of the literature search. The Web of Science database was the main source of peer-reviewed literature and this means the sources are skewed towards certain disciplines. We also excluded non-English language documents. The search terms which were used may have unintentionally excluded relevant literature because the concepts are variable and undefined, e.g., “small-scale fisheries”, or different disciplines may use different terms, e.g., “participatory monitoring”. Although we tried to address these concerns, there may be literature which was not included.

### Quantitative analysis

Once the pool of peer-reviewed CBFM documents was created, the word search tool in NVivo 12 was used to identify where papers addressed the themes of gender and participatory monitoring. The search terms that were used to identify these themes are shown in Table 1. The search terms for the theme of gender included “gender” and “women”. These were selected because papers which focus on masculinities were encompassed by the word “gender” and including the term “women” enabled us to include studies which present gender-disaggregated data but do not refer to additional aspects of gender, such as social norms and gendered division of labour. Given the wide variety of terms which can be used to describe participatory monitoring, search terms were developed using the terms outlined by Stephenson et al. (2016); see Table 1. This included papers which discussed local knowledge that was not part of a monitoring programme, so whether this knowledge was part of a monitoring activity was determined by reviewing the full text. The occurrences of the gender and monitoring search terms were used to code the nature and level of engagement of each document with these themes; see code definitions in Appendix S1. For example, whether the use of local knowledge constituted a monitoring activity.

If the document was considered to have engaged with gender and monitoring in more than one section (e.g., introduction, methods, results, discussion), it was selected for qualitative analysis. Only papers which presented case studies were selected (seven out of eight possible papers). The way in which the papers engaged with the themes was then analysed.

### Qualitative analysis

Although the literature selection and quantitative analysis methods we used are fully transparent, it is not possible to be as transparent with qualitative analysis due to the interpretive and creative processes involved in this approach (Dixon-Woods et al. 2006). In NVivo 12, the seven documents were analysed using initial coding and focused coding, based on a constructivist grounded theory approach (Charmaz 2014). This coding process was an iterative one, which involved refining the codes from the main seven documents and referring back to the results of the quantitative analysis. Throughout the analysis, memo-writing was used to develop reflexivity and explore the concepts raised through the coding process. We also referred to the pool of grey literature. This process was continued until we reached theoretical saturation. Our analysis focused on how the authors engaged with the themes of gender and monitoring in their papers, rather than the nature of the management activities or programmes described.

During this analysis we examined how authors constructed their arguments and recommendations. Rather than merely excluding or including papers, this critical approach is an essential part of theory generation (Dixon-Woods et al. 2006). However, we acknowledge that our synthesis is based on how authors present programmes and ideas, rather than on a thorough evaluation of how the research was carried out. As such, we exercise an element of trust in the authors and assume the language used in the papers is an accurate representation of their epistemological stance and research activities. Based on this analysis, several key concepts were identified regarding how gender and participatory monitoring are dealt with in the CBFM literature.

## Results and discussion

### Quantitative analysis

**Fig. 2:**
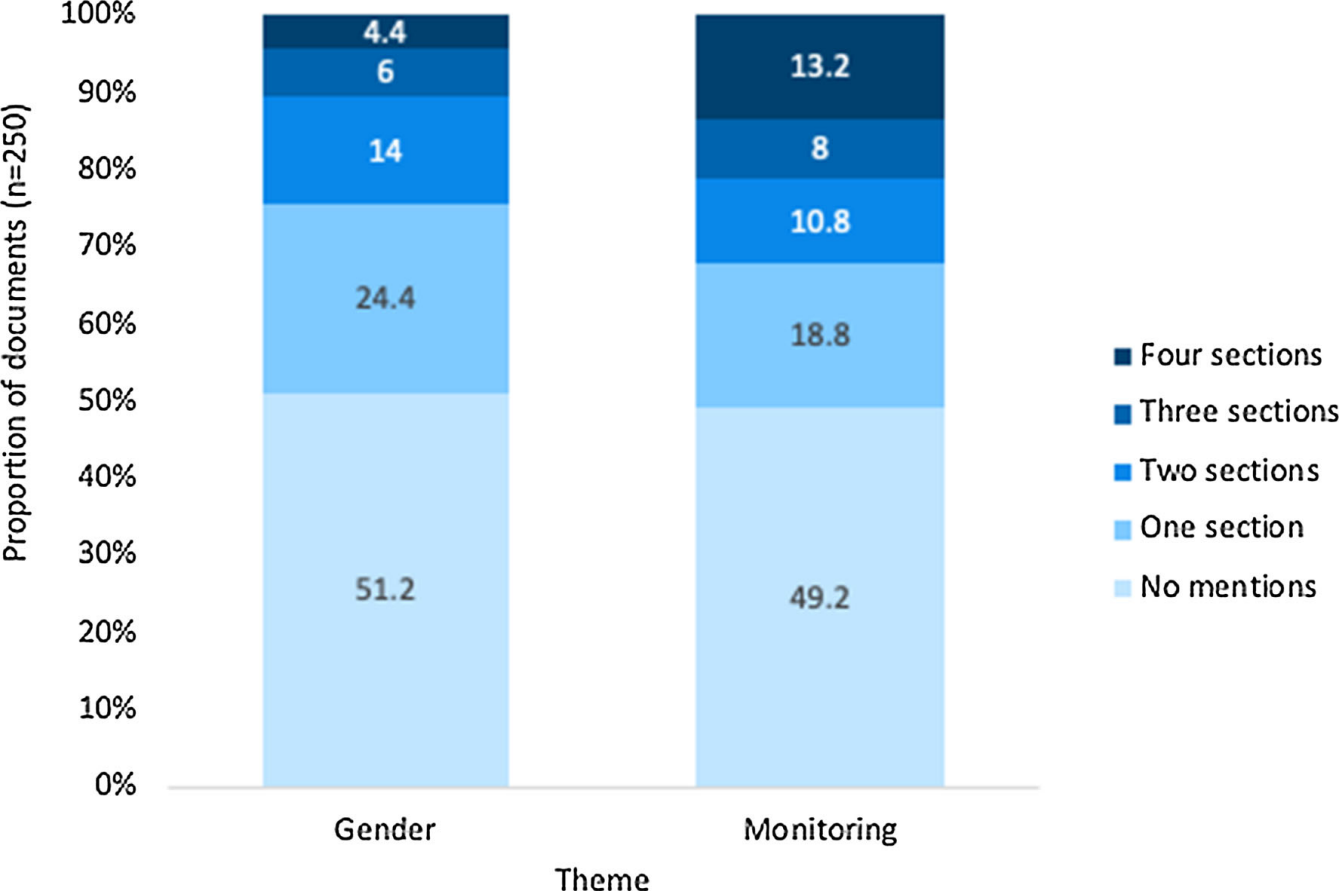
The percentage of papers which mention the themes of monitoring and gender to different extents in the main text (appendices and abstracts excluded). The sections are the introduction, methods, results, and discussion of each paper

**Fig. 3:**
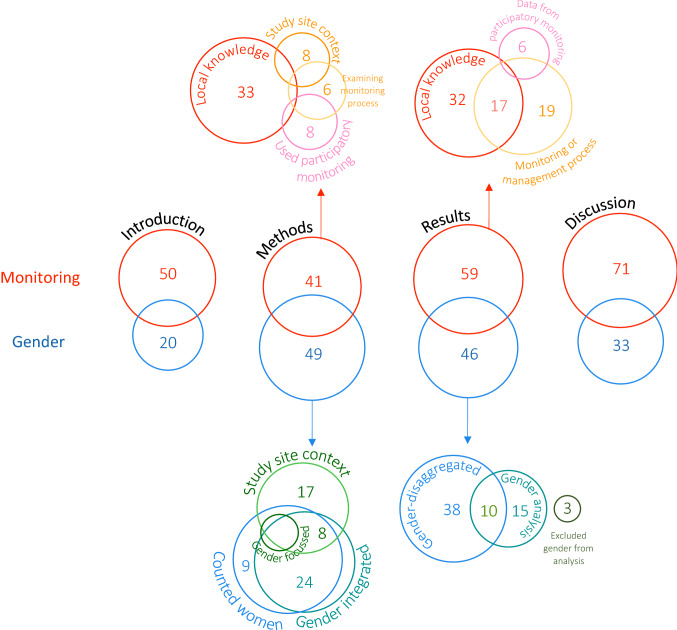
The level and nature of engagement that the 250 documents have with the themes of monitoring and gender in the main text. The size of each circle reflects the number of documents which engage with each concept in the introduction, methods, results, or discussion. Labels indicate the number of documents in each intersection. Intersections containing 5 or fewer documents were not labelled. In the methods and results sections of the papers, the engagement with the two themes in the methods and results was further broken down to reflect the way each document addressed the themes. Monitoring was mentioned in the abstract (with no mention in the main text) or appendix of 3 paper, and gender was mentioned in the appendix or project name of 4 papers

The theme of gender was mentioned in 126 of the papers and monitoring was mentioned in 130 papers (see search terms in Table 1). Both themes were mentioned in 66 papers, and 60 documents did not mention either (Fig. 1). The 48.8% of papers which mentioned gender included it to different extents (Fig. 2). We used the number of mentions of gender, and the sections of the paper which it is included in, as a proxy for the level of engagement with gender in each document. Gender was mentioned in 33 introductions, 73 methods, 66 results, and 48 discussions (Fig. 3). Of these documents, 24.4% only mentioned gender in one section of the paper and 4.4% mentioned gender in all four sections (Fig. 2). Thirty-eight papers were classified as gender-integrated, while six papers were classified as gender-focused (five papers were counted in both categories). The majority of papers which reported results relating to gender presented gender-disaggregated data (Fig. 3). Three papers included gender or counting women in the methods, only to explicitly state that there was insufficient data to include gender as a variable in the analysis.

The word search for monitoring terms (Table 1) found that 50.8% of the documents mentioned monitoring. 18.8% of the documents mentioned monitoring in one section of the study and 13.2% mentioned it in all four sections (Fig. 2). The search terms were mentioned in 63 introductions 64 methods, 79 results, and 86 discussions (Fig. 3). 32 documents focused on local knowledge in a way that was not considered to be part of a monitoring programme, i.e., mapping existing local knowledge with no mention of repeated surveying that could capture change. Forty-seven documents discussed monitoring programmes or using local knowledge in a way which constitutes a monitoring programme, these documents were included whether the term “monitoring” was used or not. The process of determining which papers engaged with local knowledge in a way that could be considered to be monitoring was challenging due to the variety of terms and framing used in the literature, and the fact that many papers referred to pilot activities which had the potential to be the baseline for a long-term monitoring programme (e.g. Paul et al. 2016).

Overall, the number of papers which engaged with each theme increased over time, with some fluctuations. In 2000–2004, the mean number of papers each year that dealt with gender was 1.6, while 1.4 dealt with monitoring. In contrast, in 2016–2020, a mean of 11.6 papers engaged with gender each year and 11.8 papers engaged with monitoring. Although this is encouraging, it should be noted that the number of papers published per year is increasing across disciplines (Herrmannova and Knoth 2016).

As shown in the blue and green circles in Fig. 3, the highest level of engagement with gender was in the methods section of the papers but the engagement with monitoring (or local knowledge) increased in the results and discussion sections. The proportion of papers which discussed both themes was relatively low, 26.4%. Breaking down the engagement with monitoring further revealed that the main focus was on local knowledge, followed by data about monitoring or management activities, and finally results of participatory monitoring programmes. The references to gender in the methods sections were divided between study site context, counting women, integrating gender, and focusing on gender. The papers which mentioned gender in the results section either presented gender-disaggregated results, gender analysis, or explicitly stated that their results or research design did not allow them to include gender in their analysis. As expected, most of the papers which mentioned gender presented disaggregated results, with fewer having a gender focus.

### Qualitative analysis

**Fig. 4:**
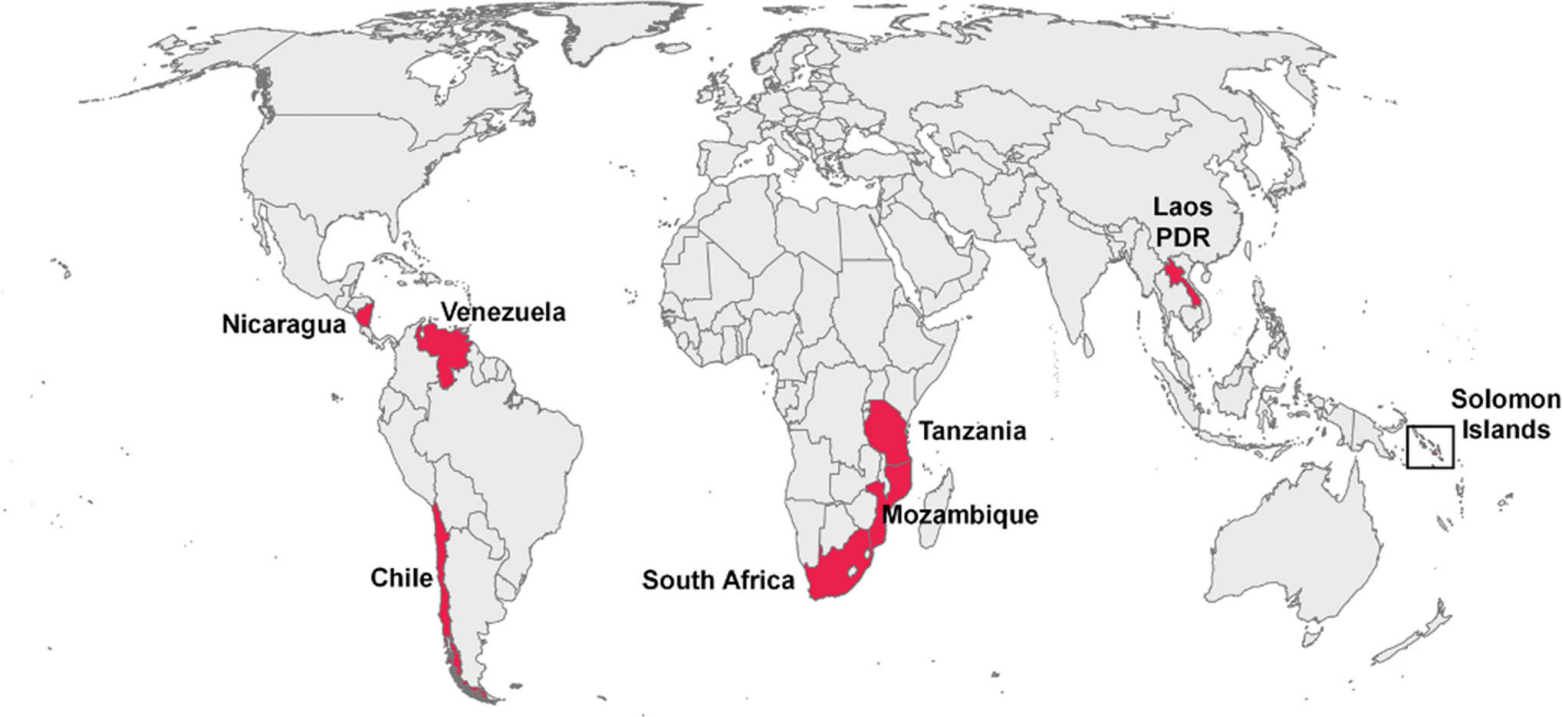
Locations of eight case studies presented in the seven papers which engaged with gender and monitoring

The seven papers selected for qualitative analysis covered a wide geographical range (Fig. 4) and a variety of fishery types (Table 2). One of the papers presented two cases (Crawford et al. 2010). Half of the cases were male-dominated, and half were female-dominated fisheries. Data collectors were majority/all women in all but one of the papers which involved implementing a monitoring programme. The remaining paper did not state the gender of the data collectors. The two other case studies did not implement a monitoring programme as part of the study and collected data from women and men (Zanetell and Knuth 2002; Gelcich et al. 2006).

**Table 2:** Papers selected for qualitative analysis and a summary of their engagement with the themes of gender and monitoring

Authors	Country	Goal/summary	Fishery and status	Dominant fisher gender	Gender	Data collectors	Monitoring
Zanetell and Knuth (2002)	Venezuela	Knowledge partnerships needed between stakeholders and case study of rapid rural appraisal. Discussion of positivist and interpretivist paradigms in CBFM	River fishery in three villages Stocks declining	Not stated, only refers to fishermen	Women and men included in data collection, but certain activities were clearly dominated by men	n/a	The RRA methods presented are not monitoring but they do advocate for integrating local knowledge and awareness of change throughout CBFM
Aswani and Weiant (2004)	Solomon Islands	Women’s community-based marine protected area implemented and evaluated using participatory monitoring, combined with a development project	Shellfish gleaning Stock declining	Women	Management designed to address women’s needs. Monitoring increased women’s involvement in management and decision-making. Women’s economic empowerment addressed	Women gleaners	In situ shellfish monitoring. Five women also trained village households to record ex situ catch data. Women’s local knowledge documented and utilised
Gelcich et al. (2006)	Chile	Evaluate co-management policy being superimposed on existing traditional CBFM	Bull-kelp harvesting (shellfish managed under same policy)	Men (some women)	Women and men interviewed and counted. Particular focus on heterogeneity in community, e.g., differences for older men, women, widows, and intracommunity dynamics	n/a	Monitoring was not a part of study methods. It was mentioned because it is part of management which is being examined
Carvalho et al. (2009)	South Africa	Community-based monitoring used to investigate sustainability of fishery. Analysed quality and reliability of monitoring data	Harder fishery Authorities aiming for fishery closure	Male	Male fishers and female data collectors. Created opportunities for women to participate in fishery and management, and increase income and skills. Data collectors had to gain fishers’ trust	Local young women	Monitoring needed to advocate for the needs of the fishing communities and enable management authorities to make evidence-based decisions
Crawford et al. (2010)	Nicaragua Tanzania	Co-management model from Fiji implemented in two other countries. Participatory monitoring was integrated in the co-management approach	Shellfish gleaning Stock declining	Women (some men)	Activities mainly involve women because harvesters are mostly women. Challenges of working with women are emphasised. Gendered aspects of decision-making not discussed	Women gleaners (some men)	Data mainly collected by gleaners monitoring the species which they normally harvest. Method was designed by the researchers to evaluate the management interventions
Paul et al. (2016)	Mozambique	Developing an accessible participatory mapping tool, which could be used for monitoring. Using the tool to map intertidal fishing grounds	Shellfish and octopus gleaning No local data, but declines in region	Women	Women collecting data on their own fishing grounds. Mitigated barriers to women’s participation. Detailed discussion of participant selection, gender norms and participant feedback. Men involved in programme	Women gleaners	Piloting a mapping tool, but this also served as the first survey with potential to repeat the data collection as a monitoring programme. Tool was developed in collaboration with participants
Patricio et al. (2019)	Lao PDR	Participatory monitoring was used to collect the data in this study. The goal of the study was to produce a baseline of catches and describe the fishery	River fishery Stock declining	Men	Discussion of gender differences in fishing activities. Recognises fisher gender as important within monitoring because of different fishing sites and gear. However, not within the scope of this study due to site selection	Gender not stated, 4 per village + student + official	Participatory monitoring used to collect data that is not available otherwise. Focus on results of monitoring, not using it for decisions in this study

These seven papers were the only case studies, from a pool of 250, which met our selection criteria, indicating that they successfully incorporated the themes of participatory monitoring and gender throughout their research. The authors demonstrated the value of participatory monitoring and explored gendered elements of monitoring or management activities. The successful implementation of these programmes provided fisheries managers and researchers with an opportunity to reflect on the authors’ experiences and to critically consider potential approaches for gender-inclusive participatory monitoring. We acknowledge the challenges of doing this work and recognise that many participatory monitoring programmes do not attempt a gender-integrated approach. Our critical reflection of these papers is in response to the valuable contribution they have made and is an effort to inform future work.

#### Reasons for engaging with gender

Our study found that relatively few CBFM papers incorporated gender throughout their research. Arguments for increasing gender equity or women’s participation are based on intrinsic or instrumental frames (Lawless et al. 2021). Intrinsic frames value gender equity for its own sake and instrumental frames see gender equity as a way to achieve other goals such as increased productivity or incomes (Tallis and Lubchenco 2014; Lawless et al. 2021). Our thematic analysis found that most papers provided both instrumental and intrinsic justifications. However, if only one frame was used, it was always the instrumental perspective. The instrumental reasons given in these papers for engaging with gender stem from recognising the value of women’s fishing activities for food security and livelihoods in the community, and the usefulness of their specialist knowledge for management. The intrinsic reasons related to human rights and changing social norms. Several researchers aimed to increase women’s participation in fisheries management over the course of their study, using monitoring as an entry point. The authors reported processes which may contribute to women’s empowerment or gender transformation, including gaining independent incomes, building self-esteem, forming women’s groups, learning new skills, and participating in fisheries management decision-making. Most of the papers problematised the lack of gender dimensions addressed in fisheries management, and four designed their studies to address this problem. The two studies which did not establish a monitoring programme (Zanetell and Knuth 2002; Gelcich et al. 2006) both collected data from women and men. Gelcich et al. (2006) pays particular attention to the role of widows in their traditional management case study. This was the only paper which highlighted the unique challenges that a specific group of women faced. The other papers referred to women more broadly. Of all the papers selected in this study, only one focused on masculinities rather than women (Siegelman et al. 2019).

#### Reasons for engaging with monitoring

Similar to engagement with gender, the motivations for establishing monitoring varied amongst the case studies, and each programme had several aims. Instrumental or intrinsic justifications were provided for participatory monitoring, where the inclusion of community members in monitoring activities was either seen as fairer (intrinsic value), or as a way to collect data or improve engagement that could benefit other management outcomes (instrumental value). In these cases, monitoring was used to evaluate management interventions (Aswani and Weiant 2004; Crawford et al. 2010), collect data in data-poor fisheries where other survey methods were not practical (Patricio et al. 2019), make evidence-based decisions (Carvalho et al. 2009; Crawford et al. 2010), build community members’ confidence and engagement in management (Aswani and Weiant, 2004), or as a tool to increase communities’ negotiating power with external actors, such as NGOs or the authorities (Carvalho et al. 2009; Paul et al. 2016). Several programmes also worked to increase the involvement of the data collectors in decision-making.

The narratives which were given about the role of data collectors or the motivating factors for participating in monitoring varied. For example, Carvalho et al. (2009) emphasised the responsibility which the data collectors shouldered and Aswani and Weiant (2004) discussed the various opportunities which were created through their programme. Some papers framed participation in monitoring as an opportunity for the participants and others emphasised the service this provided for the community and the cost this had for the participants, e.g., lost income or time burden (Aswani and Weiant 2004; Paul et al. 2016). The reasons which the authors gave for participants’ interest in the programmes were that they relied on the resources being managed, that they wanted to learn new things, or that it was a fun social activity (Aswani and Weiant 2004; Paul et al. 2016). Some papers presented the views of the participants themselves, e.g., by discussing the motivations for participating and by seeking the data collectors’ feedback about the programmes or tools, but several did not. Our analysis shows that authors characterised data collectors as instruments or agents. If data collectors were acting as instruments, they were tools being used to fulfil an existing goal, but if they were acting as agents then they were taking an active role and able to make choices and influence the system.

#### Monitoring through a gender lens

Despite the large number of papers which were identified during the literature selection, only seven engaged with both gender and participatory monitoring in more than one section of the paper. Of the six cases that implemented monitoring programmes, four of them involved women fishers monitoring the resources that they harvested, and one case involved women collecting data about men’s fishing activities. In female-dominated fisheries, monitoring was explained as a way to improve the resource which the data collectors relied on for their livelihood. In one case, having female data collectors was a way to increase women’s participation in male-dominated fisheries management decision-making (Carvalho et al. 2009). All of the case studies (other than one which did not mention the gender of fishers or data collectors explicitly) described gender differences in fishing activities or fisheries management. Patricio et al. (2019) explained that monitoring which only targets one gender is unable to quantify the catches from the whole system. In addition to accessing different habitats or target species, women and men may possess different ecological knowledge (Omoto 2004; Tawake et al. 2007).

Some cases emphasised the additional barriers to women’s participation in fisheries monitoring and management. Several authors stated that they had to adapt their way of working to accommodate women’s lower confidence, capacity, or education level (Crawford et al. 2010; Paul et al. 2016). In a community where illiteracy rates were significantly higher for women, Paul et al. (2016) developed an accessible data collection tool which used icons instead of text. Monitoring methods were also made more accessible by adopting techniques used in women’s fishing practices (Aswani and Weiant, 2004). Gender norms impacted the data collection because permission from husbands was required or male community representatives were needed to accompany the participants (Paul et al. 2016). In another case, the gender of catch-monitoring teams was shown to impact women’s willingness to participate in participatory monitoring (Sami et al. 2020).

All of the papers that mentioned women data collectors suggested that participating in monitoring was an empowering process for the women involved and provided an entry point to participation in decision-making. However, this raises the question (which several of the papers also raised) of whether this activity-specific participation of women is really translated into empowering participation (Agarwal 2001). For example, Paul et al. (2016) explored this question and showed how monitoring activities can be empowering and extractive at different points within the same programme. If women are expected to participate in monitoring without being involved in decision-making, seemingly participatory projects may serve to be gender reinforcing, rather than transformative. This raises the question of whether natural resource management activities use women’s hard work and time, without providing much benefit to the participants, under the guise of encouraging women’s participation (Buchy and Rai 2012). In these cases, participation in management is yet one more thing to do for already overburdened women. The cases included in this analysis describe various forms of benefits to women participants, e.g., income and education or fun, and acknowledge the time burden and potential lost income which participants face because of participating in the programme. In some situations, increasing women’s participation in natural resource management may do very little to change management outcomes, but women benefit from developing skills and self-confidence (Prokopy 2004). Women’s participation in fisheries management requires a nuanced approach which recognises the variation between cultures, contexts, and management approaches.

#### Knowledge valuation and prioritisation in management

One of the most common topics in the papers was the nature of fisher knowledge and participatory monitoring, how reliable it is, and the extent to which it can be integrated into fisheries management. The papers in our analysis took a variety of epistemological stances, from positivist (Carvalho et al. 2009; Patricio et al. 2019), to pragmatist (Aswani and Weiant 2004; Crawford et al. 2010; Paul et al. 2016), to interpretivist or constructivist (Zanetell and Knuth 2002; Gelcich et al. 2006). The papers which discussed implementing participatory monitoring usually included a section on assessing the reliability of the monitoring data or local knowledge, according to Western scientific standards. Due to a history of government mistrust of fishers’ catch data, Carvalho et al. (2009) made scientific reliability of the monitoring data a major part of their study. The need for their results to be accepted by the authorities required them to take a more positivist approach. Several papers took a more pragmatic approach, which used Western scientific methods but also incorporated local knowledge. However, some authors believed there was a “trade-off between community involvement and scientific rigor” (Aswani and Weiant 2004). In contrast, Zanetell and Knuth (2002) argued that fishers’ knowledge should not be seen as a lesser form of knowledge according to positivist, scientific principles. They summarised their stance by stating: “As we reconsider the paradigmatic differences between positivism and interpretivism, we suggest that expert knowledge and local knowledge not be viewed as metaphorical boxers contending for a world title, but rather as teammates jointly contributing to our understanding of and capacity to live responsibly and sustainably in our world” (Zanetell and Knuth 2002:p810). The papers which used an interpretivist approach did not seek to validate the views of community members with scientific data, rather they showed how such information could be used to develop more sustainable management approaches. The epistemological stance taken in each case reflected the target audience of the research, with research aimed at government authorities taking a more positivist approach and research aimed at local communities taking a more interpretivist approach.

**Fig. 5:**
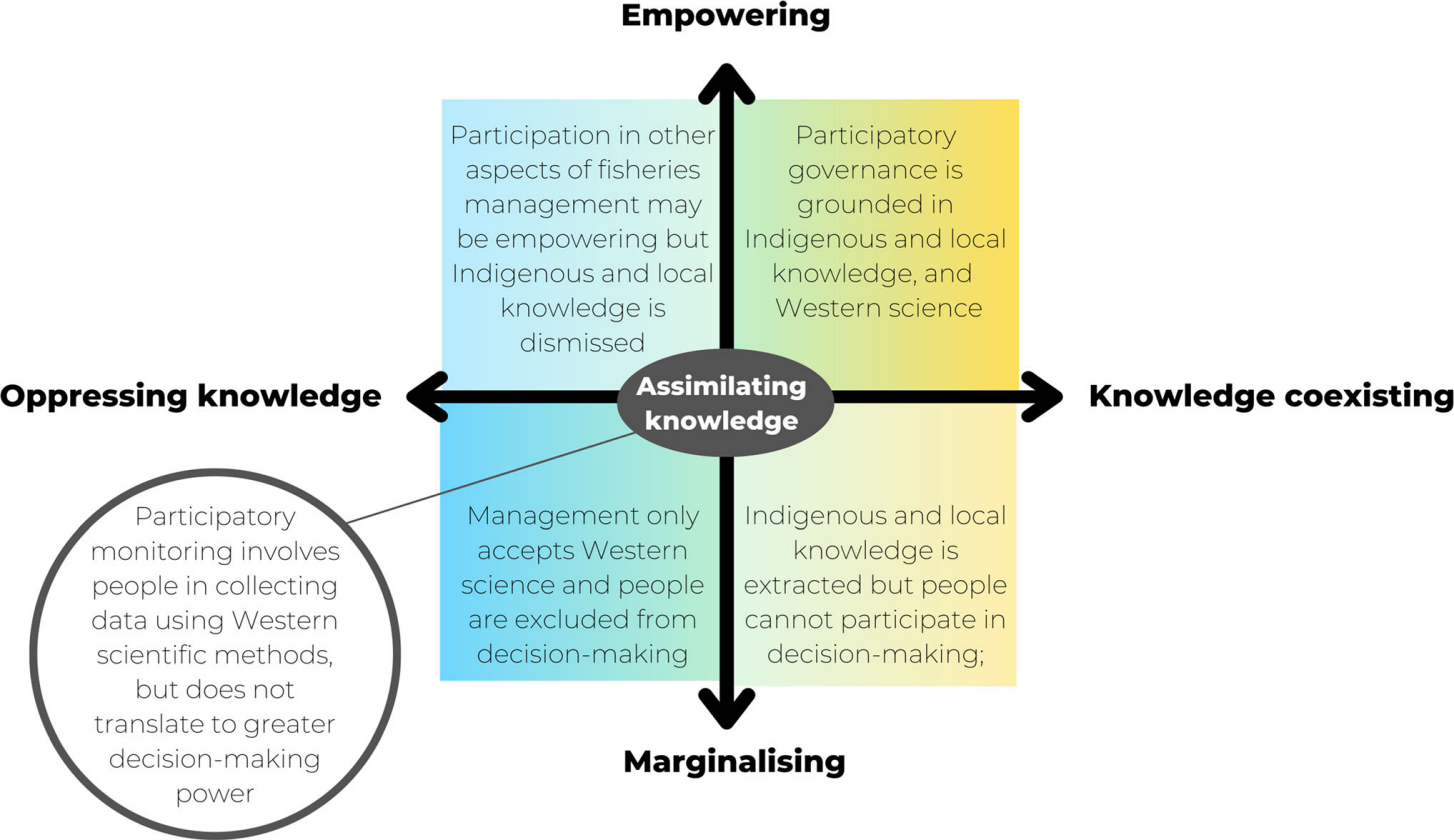
Fisheries management activities can result in people and knowledge becoming oppressed/marginalised or accepted/empowered. The X axis represents a continuum of knowledge integration from Indigenous and local knowledge being oppressed to all forms of knowledge coexisting as equals (adapted from Reid et al. 2020). The Y axis indicates the form of participation which exists in a management system, based on the decision-making power of marginalised people (adapted from Agarwal 2001). The text provides example scenarios which might occur at different location in this framework

The papers considered various forms of information which were collected by fishers, community members, or researchers, and were used for management decision-making or for research. The approaches that were used to collect this information involved varying degrees of fisher participation. Figure 5 demonstrates that there are two dimensions which should be considered in participatory monitoring: the nature and participation of people in the programme (as shown on the Y axis); and the ways in which different knowledges are treated (X axis).

Many papers discussed “incorporating” or “integrating” fisher knowledge into fisheries management. However, they were not clear about what this means. For example, were they talking about appropriating local knowledge into the existing dominant system (Reid et al. 2020)? Or were they advocating for governance regimes which are designed to accept and expect the fishers’ knowledge, and that view data collected via participatory monitoring as part of the “best available” information (Stephenson et al. 2016)? This perspective was illustrated by the way monitoring programmes were established. Rather than co-designing monitoring programmes in conjunction with developing participatory governance regimes that are developed to meet the needs of fishing communities, it is common for participatory monitoring programmes to begin with external actors (e.g., researchers or NGOs), training community members to carry out an established scientific protocol which was developed without their input. This reflects the emphasis on collecting data which can be considered rigorous and valid, according to Western scientific standards. Reid et al (2020) argued that fisheries research and management must move beyond “knowledge integration”, which has been used to resolve the dichotomous view of Western science and Indigenous knowledge (Nadasdy 1999), and cultivate an ethic of knowledge coexistence. Figure 5 shows different levels of participation (Agarwal 2001) and different ways of relating to local knowledges in management could create various scenarios. Depending on the aim of a particular study, different epistemological perspectives may be appropriate. Nevertheless, it is important to consider how these perspectives could be influencing power dynamics in a monitoring programme, i.e., between the researchers and the participants, between groups within a community, or between the community and external actors.

Furthermore, using the common terms of “incorporating” or “integrating” fisher knowledge into fisheries management makes it easy to overlook the people who create and own this knowledge. The shift towards integrating local knowledge into fisheries management is only required because Indigenous knowledge, fisher’s experiential knowledge, and other traditional management activities have been devalued and dismantled due to colonisation (Johannes 2002). For example, Hau’ofa (1993) demonstrates how the colonial and “belittling view” of Pacific Island people and societies that has often been propagated by social scientists and internalised by many Islanders, including himself at times, is connected with a colonial view of islands as small, poor, and constrained by their land area, rather than defining them based on their rich connection with vast oceans. All of the case studies in the qualitative analysis were located in countries which had been colonised but are now independent. Calls for knowledge assimilation, which focus on the benefit Indigenous knowledge can bring to Western scientific fisheries management, serve to separate knowledge from the people and the culture that constructed it. This “systematic disregard of marginalised people's local and historical worldviews renders most forms of local knowledge oppressed. Oppressed knowledge is, in turn, more difficult to mobilise on behalf of the construction of new knowledge” (Deshler and Grudens-Schuck 2000, p. 598). Although it has been an important step, researchers need to move past proving or justifying fisher knowledge and participatory monitoring according to Western research paradigms and colonial management practices. For example, a participatory monitoring programme in Kiribati and Vanuatu used participatory monitoring “to catalyse and support community-led conversations and to bridge worldviews of community members to those of national agencies and their partners” (Andrew et al. 2020). Hau’ofa (1993, 1998) demonstrates how a strong and optimistic worldview can shape how people see themselves, their environment, and their knowledge, through his advocacy for a new view of Oceania, linking themes of regional identity, personal empowerment, decolonisation, and custodianship of natural resources. Changing how we view, use, and talk about these forms of knowledge, and the people who construct them, can contribute to reconciliation or decolonisation.

Several papers applied the deficit model to participatory monitoring or focused on proving the reliability of participatory monitoring. The deficit model is the perspective that fisher knowledge or participatory monitoring is inherently lacking compared to information created using Western/dominant scientific methods. None of the case studies in the qualitative analysis were dismissive of local knowledge but some did discuss a view —that they described as being widely held—that fisher knowledge and scientific knowledge are dichotomous or incompatible. In contrast to that view, some papers in this study framed these different forms of knowledge as complementary and valuable. However, the emphasis on proving the reliability of fisher knowledge and participatory monitoring in the literature plays a useful role in showing the value of these types of information in grounding management decisions in evidence, especially in data-poor fisheries (Hind 2014). It also demonstrates the perception amongst researchers that participatory monitoring and local knowledge must be justified according to positivist /dominant research paradigms to be considered usable. Throughout this paper, and in many of the papers identified in this study, authors referred to monitoring as a scientific data collection process which has been recently established. However, almost every case mentioned the concerns that communities have about their declining resources. This concern is the result of direct, ongoing observation of the resources, produced by Indigenous knowledge and/or local resource users. However, this does not seem to be considered “monitoring”. Western scientific standards often limit the data, knowledges or methods which are recognised, but Indigenous scholars continue to develop methodologies which move beyond these constraints and produce new insights and transformation, and which work toward social justice (Smith 2006; Wilson 2008; Smith et al. 2016).

#### Replicability and transparency

Given that many of these programmes were pilots, transparency and repeatability of the methods is necessary so that they can be repeated or used elsewhere. Our analysis found that the methods for data collection were reported more clearly than the methods for community-engagement or participant selection. In some of the cases, it was not possible to tell who was participating in particular activities or how many people were involved. However, some papers did include descriptions of participant selection criteria, relevant social norms, and methods used to build trust with participants. All of the papers discussed training activities involved in establishing the monitoring programmes. These papers often argued for increased community participation or gender inclusion in fisheries management, so it would be appropriate for them to explain how they approached these issues in their own studies more clearly. Documenting the challenges of conducting this type of work would be valuable to other practitioners and researchers. An excellent example is Paul et al. (2016) who described their participant selection process in detail, included feedback from the participants about the methods, and reflected on the relationship between the participants and the researchers. We acknowledge that it might not be practical to document this level of reflexivity in all studies, and that researchers may be discouraged from exploring why their approaches to improving gender equity or increasing community participation were successful or not. They may fear criticism but reflecting on these aspects of the research is necessary to avoid reinforcing the very problems researchers are trying to solve (Barnaud and van Paassen 2013). Furthermore, journals and peer reviewers should incentivise this by requiring authors to show such reflexivity in their papers, and supporting them to do so.

#### Marginalisation narratives

The way in which these authors described participatory monitoring and gender inclusion often framed them both in terms of power dynamics. The power dynamics which were discussed in these papers include intracommunity dynamics and dynamics between communities and external actors. Various characteristics were cited as influencing these dynamics: gender; race; migration history; small-scale fishers vs commercial fishers; socio-economic status; and users of traditional management. Most of the papers in this study focused on the marginalisation of small-scale fishing communities and the additional barriers faced by women. However, the nature of these narratives varied depending on the local context. For example, Carvalho et al. (2009) designed a monitoring programme in South Africa to enable fishers, who were marginalised under apartheid and neglected during the subsequent fisheries reform, to assert themselves against the management authorities. However, they also recognised the gender dynamics which exist within those fishing communities and designed a monitoring programme which would serve to increase women’s participation whilst addressing their other goals.

The papers focusing on gender framed women as a marginalised and vulnerable group and much of the participatory monitoring literature viewed small-scale fishers the same way, with a few papers specifically focusing on fisherwomen. The papers selected in this study often referred to small-scale fishers, women, Indigenous people, or youth as marginalised. Marginalised people live diverse, meaningful, and interesting lives and an array of methodologies have been developed to represent their experiences authentically (Smith 2006). Intersectionality provides a valuable framework to examine how the overlapping identities of people within fishing communities may influence the power dynamics that shape decision-making. Our findings show that this complexity is often overlooked by researchers. Prime examples of this are the ways in which the words “community” and “women” are regularly used to refer to single homogenous groups. Only one of the studies in the qualitative analysis addressed the specific challenges faced by one group of women, rather than referring to women as a single group. Treating women or communities as homogenous enabled authors to emphasise the conflict or power differential between the community and external actors, or between women and men. This may serve to emphasise a relevant concern, but it also erases the rich and diverse identities which exist within these groups, and the intracommunity dynamics which ensue (Lau et al. 2021).

The papers in this analysis viewed CBFM or participatory monitoring as a potential way to change power dynamics, but this assumes that participation translates into a meaningful change in power. Some of the papers focused on giving the community as a whole more power, whilst others focused on specific groups, i.e., fishers or women. If the goal is to genuinely change the power dynamics, then critical consideration of the ways these changes may impact different types of people is needed. An intersectional approach enables us to move away from the male–female binary view of gender and the idea of women as a static and homogenous group (Ravera et al. 2016a, 2016b). Programmes must be culturally responsive and context-dependent to provide the nuance which this requires.

## Conclusion

Although research on CBFM is becoming more gender-aware and acknowledging the value of participatory monitoring, a thorough analysis of how these themes are portrayed in the literature shows that a more nuanced understanding of the issues is needed. Improved understanding of these themes is needed at the stage of designing research or management programmes and when presenting methods, research findings, or programme outcomes. Research projects, and the papers which present them, often address specific objectives, so data is included or excluded based on whether it strengthens the researcher’s core argument. This literature review highlights some potential biases and elucidates some of the steps which researchers can take to navigate these concerns more effectively.

This study did not evaluate the programmes which were presented in the case studies, rather we examined how authors presented their work. Although future study is needed to explore these research questions in situ, we recommend:

1. Themes of participation and gender should be considered during project design, not merely as an afterthought. Currently, a high proportion of papers include a brief mention of the themes but do not integrate them into the methodology and research design. If we are to enact the principles outlined in the SSF guidelines (FAO 2015), a more strategic approach to these themes is needed. In particular, the principles of respect of cultures, which recognises the “traditional and local knowledge and practices” of fishing communities (principle 2), gender equality and equity (principle 4), equity and equality (principle 5), and consultation and participation (principle 6).
2. A more transparent and reflexive approach is needed for designing, evaluating, and reporting of CBFM activities. Researchers should critically reflect on the power dynamics and social processes occurring in their work, as presenting these issues would enable other researchers to improve their own study design. The peer review process should encourage such honesty and vulnerability, rather than considering it to be a poor reflection on the research.
3. The social aspects of methods should be reported with as much clarity as technical methods. Transparency and replicability are required for technical methods but are consistently lacking with regards to the social aspects of CBFM research. For example, some studies do not report the number of participants or include gender-disaggregated data. We recommend that participant selection and community-engagement methods should be included with as much detail and transparency as afforded ecological data collection methods.

These recommendations seek to promote more ethical, meaningful research that is less likely to reinforce existing power imbalances or disregard the importance of adapting to the local context. For researchers to follow these recommendations, peer reviewers must accept this critical reflexivity, rather than consider it a limitation. Peer reviewers must demand transparency and replicability in every aspect of the research. Management approaches which aim to increase community or women’s participation in resource management may have the potential to become empowering processes for fishing communities. However, managers and researchers must critically consider these issues to realise this vision.

## Supplementary Information

Below is the link to the electronic supplementary material.

Supplementary file1 (PDF 712 kb)
