# Supplementary material for: Participatory monitoring in community-based fisheries management through a gender lens
Source: Ambio. 2022 Sep 20;52(2):300–18. doi: 10.1007/s13280-022-01783-3 (PMC9755429; doi:10.1007/s13280-022-01783-3)
Supplement: Supplementary file 1 — Supplementary file1 (PDF 712 kb) [file 13280_2022_1783_MOESM1_ESM.pdf]

Ambio

Supplementary Information
This supplementary information has not been peer reviewed.

Title: **Participatory monitoring in Community-Based Fisheries Management through a gender lens**

Appendix S1: Codes applied in quantitative analysis

Theme	Code	Paper section	Definition
Monitoring & Gender	Study site context	Methods	Monitoring or gender is mentioned when describing study sites or existing management.
Monitoring	Local knowledge	Methods & results	Local knowledge is collected as part of the methods or presented in results section.
	Used participatory monitoring	Methods	Participatory monitoring was used to collect the data for the study.
	Examining monitoring process	Methods	Data is collected about the monitoring programme activities, participants or how the monitoring is used in management.
	Data from participatory monitoring	Results	Data and findings from participatory monitoring are presented.
	Monitoring or management process	Results	Analysis or evaluation of the monitoring activities, or how the data are used for management, is conducted.
Gender	Women counted	Methods	Gender of participants is recorded, or data is collected about the number of women and men doing different activities e.g., members of groups or fishing
	Gender integrated	Methods	Data is collected about various topics in a way that includes women and men, e.g., separate focus group discussions, or programme design includes women and men
	Gender focused	Methods	Gender is the main focus of the research and data is collected on a variety of aspects of gender, e.g., social norms, institutions, or power dynamics
	Gender-disaggregated	Results	Quantitative or qualitative gender-disaggregated data is presented
	Gender analysis	Results	In depth analysis is presented, which explores more nuanced aspects of gender, e.g., social norms, institutions, or power dynamics
	Excluded gender from analysis	Results	Papers specifically stated that gender could not be included in analysis due to insufficient data or other constraints
